# A ligation-based single-stranded library preparation method to analyze cell-free DNA and synthetic oligos

**DOI:** 10.1186/s12864-019-6355-0

**Published:** 2019-12-27

**Authors:** Christopher J. Troll, Joshua Kapp, Varsha Rao, Kelly M. Harkins, Charles Cole, Colin Naughton, Jessica M. Morgan, Beth Shapiro, Richard E. Green

**Affiliations:** 1 Claret Bioscience LLC, Santa Cruz, CA 95060 USA; 2 Department of Ecology and Evolutionary Biology, University of California, Santa Cruz, CA 95064 USA; 3 Department of Biomolecular Engineering, University of California Santa Cruz, Santa Cruz, CA 95064 USA; 4 Howard Hughes Medical Institute, University of California Santa Cruz, Santa Cruz, CA 95064 USA

**Keywords:** SRSLY, Single-stranded library, Next-generation sequencing, Cell-free DNA, Oligos, Nucleosome positioning

## Abstract

**Background:**

Cell-free DNA (cfDNA), present in circulating blood plasma, contains information about prenatal health, organ transplant reception, and cancer presence and progression. Originally developed for the genomic analysis of highly degraded ancient DNA, single-stranded DNA (ssDNA) library preparation methods are gaining popularity in the field of cfDNA analysis due to their efficiency and ability to convert short, fragmented DNA into sequencing libraries without altering DNA ends. However, current ssDNA methods are costly and time-consuming.

**Results:**

Here we present an efficient ligation-based single-stranded library preparation method that is engineered to produce complex libraries in under 2.5 h from as little as 1 nanogram of input DNA without alteration to the native ends of template molecules. Our method, called Single Reaction Single-stranded LibrarY or SRSLY, ligates uniquely designed Next-Generation Sequencing (NGS) adapters in a one-step combined phosphorylation/ligation reaction that foregoes end-polishing. Using synthetic DNA oligos and cfDNA, we demonstrate the efficiency and utility of this approach and compare with existing double-stranded and single-stranded approaches for library generation. Finally, we demonstrate that cfDNA NGS data generated from SRSLY can be used to analyze DNA fragmentation patterns to deduce nucleosome positioning and transcription factor binding.

**Conclusions:**

SRSLY is a versatile tool for converting short and fragmented DNA molecules, like cfDNA fragments, into sequencing libraries while retaining native lengths and ends.

## Background

For high-throughput sequencing, DNA molecules must be converted into sequencing libraries, which requires ligation of sequencer-specific adapters [1]. Conventional methods for Next-Generation Sequencing (NGS) library preparation convert only double-stranded DNA (dsDNA) into library-ready molecules. Prior to adapter ligation, conventional dsDNA protocols perform end-polishing, which blunts the termini of each template molecule by using DNA polymerases to fill in 5-prime overhangs and digest 3-prime overhangs. In most cases, an additional polymerase will A-tail the 3-prime ends of template DNA to promote efficient ligation of the sequencer-specific adapters [2, 3]. While end-polishing is a prerequisite for efficient dsDNA NGS adapter ligation, it renders all molecules uniformly blunt, obscuring the native termini of molecules and changing their true lengths. Furthermore, conventional dsDNA methods are unable to convert single-stranded DNA (ssDNA) or dsDNA nicked on both strands into sequencer compatible molecules. A variation of conventional dsDNA NGS library preparation uses Tn5 transposase to both cleave the DNA template and deliver adapters [4]. While not dependent on end-polishing or adapter ligation per se, transposase-based methods also fail to capture the native termini of molecules or convert ssDNA and nicked dsDNA into library molecules.

Single-stranded DNA library preparation methods offer several advantages over traditional dsDNA methods [5–7]. By denaturing the duplexed template DNA prior to adapter ligation and maintaining the DNA as single strands through at least an initial adapter ligation, single-stranded preparation methods are theoretically able to convert all of the molecules captured by traditional dsDNA library preparation methods as well as nicked dsDNA and ssDNA molecules. Originally developed for the genomic analysis of highly degraded ancient DNA [7, 8], ssDNA library preparation methods have been adopted for other fragmented sample types such as such as cell-free DNA (cfDNA) and DNA purified from Formalin Fixed Paraffin Embedded (FFPE) sections, due to their efficiency in converting a high fraction of input DNA fragments into sequencing library molecules and their ability to capture small DNA fragments. Further, the sequencing reads from some ssDNA library methods represent the natural 5-prime and 3-prime ends of the input DNA fragments. Thus, when mapped to a reference genome, these data reveal the exact genomic location of the input fragments; an important feature for cfDNA researchers studying biological fragmentation patterns.

Cell-free DNA found circulating in blood plasma and other bodily fluids contains a wealth of biomedical information that can be assayed by NGS with a minimally-invasive blood draw. A number of studies and commercial offerings use NGS data obtained from blood plasma-derived cfDNA to monitor prenatal health, organ transplant reception, cancer detection and progression, and other diseases [9–14]. In healthy individuals the vast majority of cfDNA recovered from blood is thought to originate from apoptotic lymphoid and myeloid cells, with a limited number of fragments deriving from other tissues [12, 15, 16]. However, during pregnancy or disease progression, studies have shown that blood plasma may also contain DNA fragments derived from e.g. fetal or tumor cells undergoing apoptosis, necrosis, or other forms of cell death [12, 17–21].

The length distribution of DNA extracted from blood plasma is centered around 167 base-pairs (bp). Thus, cfDNA fragments are thought to be mono-nucleosomal, the result of chromatosome (histone octamer core, also known as the nucleosome core particle, and an associated linker histone) imparted protection from nuclease degradation [12, 15, 16, 22–24]. In addition to DNA fragments centered around 167 bp, cfDNA also contains shorter DNA fragments (< 100 bp) that may not derive from nucleosome-bound DNA. Recent studies examining cfDNA within this smaller, sub-nucleosome size range show that these fragments may be the result of nuclease protection by other DNA binding proteins, such as transcription factors. Other components of cfDNA can include mitochondrial DNA and microbial DNA [16, 22, 25].

Several single-stranded library preparation methods have been described since 2013 [8, 22, 26–30]. However, widespread adoption by the NGS community has been hindered by the fact that they are more time consuming and require more enzymatic steps than traditional dsDNA methods. In addition, some ssDNA methods require exotic or expensive reagents [22, 26] and many necessitate the use of primer extension to create a second strand to facilitate sequence adapter ligation [8, 26, 28–30]. Also, in some cases special bioinformatic processing of the data is required to deal with artifacts introduced as a consequence of library prep [22, 28].

Here we describe a fast, simple, and efficient ligation-based single-stranded DNA library preparation method engineered to produce complex NGS libraries from as little as one nanogram (ng) of input DNA without altering the native ends of template molecules. Our method, called Single-Reaction Single-stranded LibrarY or SRSLY, requires no exotic reagents and can be completed in 2.5 h. SRSLY works by ligating uniquely designed NGS adapters in a single combined phosphorylation/ligation reaction without requiring end-polishing. Both SRSLY adapters are modified from the splint-adapter design introduced by Gansauge *et al*^26^. The approach of splint-ligation of both adapters was introduced by the SPLAT method, developed for bisulphite sequencing [26, 27]. SRSLY builds on these features with a streamlined workflow, a robust adapter design, and an optimized single-step ligation scheme that efficiently delivers both adapters.

We present standard sequencing metrics produced by SRSLY libraries made with cfDNA from healthy human donors and compare our results to those of commercially available library preparation methods. We then highlight the benefits of ssDNA libraries generated using SRSLY compared to dsDNA preps using synthetic duplexed oligonucleotides. Next, we demonstrate the ability of SRSLY to capture short length ssDNA fragments, and the ability to assay oligonucleotide purity using single-stranded synthesized oligos of varying length and known sequence. Finally, we demonstrate how SRSLY libraries empower fragmentomic analyses of cfDNA data by capturing a wide range of DNA fragment lengths without altering their native 5-prime and 3-prime termini. Given its efficiency and ease of use, SRSLY is a drop-in replacement for both ssDNA and dsDNA library preparation methods for many applications.

## Results

### Library construction

**Fig. 1:**
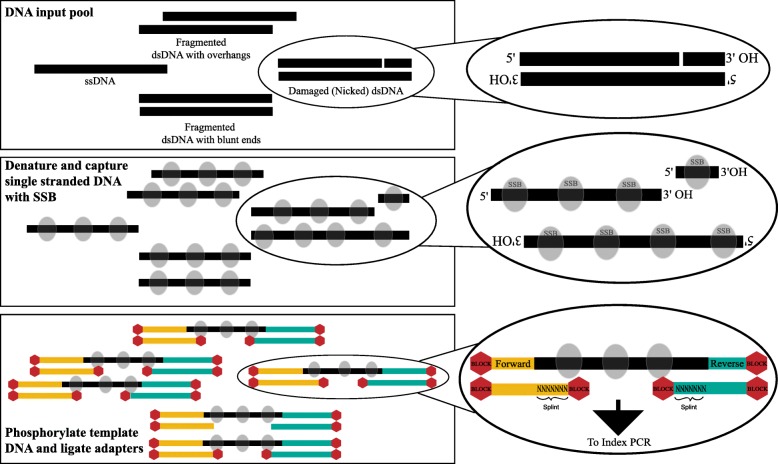
Schematic overview of SRSLY. A DNA input pool of diverse template molecules is denatured with heat and maintained as single-stranded molecules through a cold-snap and use of a thermostable single-stranded DNA binding protein (SSB). Template DNA is phosphorylated and SRSLY splint adapters are ligated in a combined phosphorylation/ligation reaction. Adapters contain a random single-stranded splint overhang and ligation blocking modifications on all termini except for the ones that facilitate correctly oriented library molecules. After clean up, molecules are ready for index PCR

The SRSLY method creates Illumina sequencing libraries from fragmented or degraded template (input) DNA (Fig. 1). Template DNA, which can be a complex mixture of dsDNA, ssDNA, and nicked dsDNA, is first heat denatured and then immediately cold shocked in order to render all template DNA molecules uniformly single-stranded. The DNA is maintained as single-stranded throughout the ligation reaction by the inclusion of a thermostable single-stranded binding protein (SSB). Next, the template DNA, which is now uniformly single-stranded and coated with SSB, is placed in a phosphorylation/ligation dual reaction with directional dsDNA NGS adapters that contain single-stranded overhangs.

Both the forward and reverse sequencing adapters share similar structures but differ in which termini is unblocked in order to facilitate proper ligations. Both sequencing adapters are dsDNA, except for a single-stranded splint overhang of random nucleotides that occurs on the 3-prime termini of the bottom strand of forward adapter and the 5-prime termini of the bottom strand of the reverse adapter. In this way, the forward (P5) Illumina adapter is always delivered to the 5-prime end of template molecules and the reverse (P7) Illumina adapter is always delivered to the 3-prime end of template molecules. Thus, the native polarity of all input DNA molecules is retained.

During the dual phosphorylation/ligation reaction, T4 Polynucleotide Kinase (PNK) prepares template DNA termini for ligation by phosphorylating 5-prime termini and dephosphorylating 3-prime termini. T4 PNK works on both ssDNA and dsDNA molecules and has no activity on the phosphorylation state of proteins [31–33]. Simultaneously, the random nucleotides of the splint adapter anneal to the single-stranded template molecule. This creates a short, localized dsDNA molecule, enabling ligation of template to adapter with T4 DNA ligase, which has high ligation efficiency on double-stranded DNA templates but low efficiency on ssDNA [34]. After the single phosphorylation/ligation reaction is complete, the library DNA is purified and placed directly into standard NGS indexing PCR, compatible with both traditional single or dual index primers.

### Performance of the SRSLY protocol

To evaluate the quality and quantity of data produced by SRSLY we generated several sequencing libraries from two plasma cfDNA extracts obtained from two healthy human individuals (H-69 and H-81, respectively) using SRSLY, two standard commercially available end-polishing dsDNA library kits (New England Biolabs® NEBNext® Ultra™ II and TaKaRa SMARTer® ThruPLEX® Plasma-Seq) and a popular commercially available ssDNA library kit (Swift Bioscience Accel-NGS® 1S). After library preparation and quantification, libraries were paired-end sequenced on Illumina HiSeq X (2 × 150 bp) to roughly 400 million read pairs per cfDNA extract for SRSLY and NEBNext Ultra II and to roughly 100 million reads pairs per cfDNA extract for TaKara SMARTer and Swift 1S. Sequencing data from libraries generated from the same cfDNA extract and library preparation method were combined for analysis. We merged the forward and reverse sequence reads when these reads overlap to generate single reads representing the original DNA fragment. Since the majority of sequence reads from cfDNA are about 167 bp long, only merged reads (where read 1 and read 2 overlap by at least 30 bp of complementarity) were used for downstream analyses. Additional files 1,2 contain the sequencing metrics for all sequenced libraries. The data generated resulted in about 15-fold coverage of the human genome for both SRSLY and NEBNext Ultra II samples per cfDNA extract and about 5-fold coverage for both TaKaRa SMARTer and Swift 1S per cfDNA extract.

**Fig. 2:**
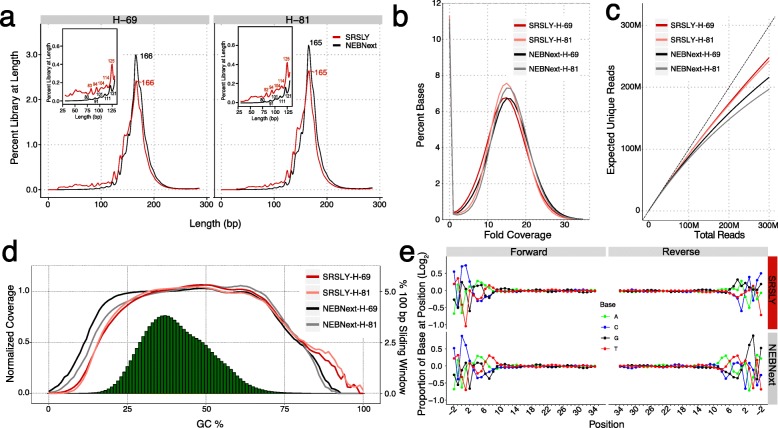
Standard NGS metrics for merged reads from SRSLY and NEBNext Ultra II libraries from healthy human cfDNA extracts H-69 and H-81. Unless otherwise stated, all libraries for each method were combined by cfDNA extract prior to analysis and filtered for PCR duplicates and a quality score equal to or greater than q20. (**a**) Insert distribution plots for cfDNA extracts H-69 and H-81, respectively. (**b**) Fold coverage by base percent across the human genome (*hg19*) for SRSLY and NEBNext by cfDNA extract. Combined libraries were subsampled to similar read depth prior to fold coverage calculations. Subsampled depth was set at 295 M reads, the limit of sequenced reads for SRSLY-H-81. (**c**) Preseq complexity estimate for SRSLY and NEBNext by cfDNA extract. Three libraries of equivalent sequencing depth per method were combined to estimate complexity, since more libraries were made via SRSLY than NEBNext. Files containing the PCR duplicate reads were used to facilitate complexity estimates (**d**) Normalized coverage as a function of GC content over 100 bp sliding scale across the human genome for SRSLY and NEBNext by cfDNA extract. Green histogram represents the human genome GC across the 100 bp sliding window. (**e**) Normalized, log-transformed base composition at each position of read termini starting 2 bp upstream and extending to 34 bp downstream of read start site for combined cfDNA extracts for SRSLY and NEBNext. All reads regardless of insert length considered

As expected, libraries generated by SRSLY and NEBNext Ultra II cfDNA have length distribution features typical of cfDNA fragments. They both show fragment length distributions centered around the chromatosome length at 167 bp. They both show the sawtooth pattern in shorter fragments that are the result of DNase I cleaving the exposed minor grove of nucleosome bound DNA at a periodicity of 10.4 bp^35^ (Fig. 2a, Additional file 3). However, as shown in Fig. 2a and its inset, the two preparation methods differ in the proportion of reads captured at different fragment lengths, as well as the length distribution of the sub-peaks present in the sawtooth pattern. SRSLY libraries have a higher abundance of shorter, i.e. sub-nucleosome length, reads with shorter sub-peaks in the sawtooth pattern versus NEBNext Ultra II. These observations are hallmark features of ssDNA preparation methods [16, 22]. The increased proportion of sub-nucleosome-sized reads reflect the increased ability of ssDNA methods to convert short and/or nicked DNA fragments into sequence library molecules. The difference in sub-nucleosome peak sizes is likely due to the ability of SRSLY to retain native termini compared to dsDNA methods. In dsDNA library methods, 5-prime overhangs are filled in and 3-prime overhangs are removed. Thus, the observed length of a given DNA molecule will be dependent on what type of overhangs are present. This information is lost during the end-polishing step required in dsDNA library preps.

We compared the read coverage, estimated complexity (number of unique molecules in the library), and GC content of SRSLY versus NEBNext Ultra II libraries for both cfDNA extracts. Figure 2b shows that SRSLY produces fold-coverage similar to that of the NEBNext Ultra II kit and that both methods produce relatively uniform genomic coverage. Figure 2c shows that at a sequencing depth of 300 million reads, or roughly one HiSeq sequencing lane, SRSLY libraries are estimated to have higher molecular complexity than NEBNext Ultra II libraries. This difference might be a reflection of SRSLY’s ability to recover nicked and ssDNA strands lost to traditional dsDNA library preparation. Figure 2d shows that the GC content of SRSLY libraries is similar to that of the NEBNext Ultra II kit. The GC content plots for both SRSLY and NEBNext Ultra II are shifted towards GC rich regions compared to the human genome reference (histogram, plotted in green) because cfDNA is biologically enriched for GC-rich regions [35]. The differences shown in regions of low GC content between SRSLY and NEB Ultra II could be either the result of reaction conditions or differences in polymerases used during index PCR.

Most dsDNA library preps, including NEBNext Ultra II, perform end-polishing on the input DNA molecules. Because the SRSLY prep delivers the sequencing adapters to the native termini of DNA fragments, we can examine the base composition at and around the exact 5-prime and 3-prime end of each DNA fragment with single nucleotide resolution. Note that the end-polishing procedure retains the native 5-prime end of molecules. However, the 5-prime overhang “fill-in” and the 3-prime overhang exonuclease activity of T4 DNA polymerase generates a 3-prime end that is not representative of the original molecule when overhangs of either type are present. In this way, the end-polishing procedure is expected to make all 3-prime ends mirror what is present at the 5-prime end of the complementary strand.

To test these expected differences in DNA termini information, we compared the base composition per position across the start coordinates for both the forward (read 1) and reverse (read 2) reads, inferred from the merged read dataset, for both the SRLSY and the NEBNext Ultra II cfDNA libraries (Fig. 2e). There are four notable findings. First, for both SRSLY and NEBNext there is significant deviation from the average base composition at the start of each read, as well as upstream of the biological fragmentation point. This is a well-documented feature of the cfDNA nucleosome protection model [16, 36, 37], further discussed in the cfDNA results section below. Second, unlike the dsDNA library data, the average base composition for the start of the forward reads and the start of reverse reads differ in SRSLY libraries. This suggests that cfDNA fragments often contain overhangs that are altered during the end-polishing steps of dsDNA library prep. Third, the average base composition for the start of the forward read in NEBNext Ultra II libraries are exactly the reverse-complement of the average base composition for the start of the reverse read. This is expected for molecules that are uniformly blunt ended, the byproduct of end-polishing during dsDNA library preparation. Finally, the average base composition for the start of the forward read in SRSLY libraries is nearly identical to that of NEBNext Ultra II libraries. This is the expected result as end-polishing retains the native 5-prime ends, as does the SRSLY direct ligation procedure.

We compared the length distribution, read coverage, complexity, GC content, and DNA termini results of the SRSLY prep to those of the TaKaRa SMARTer and Swift 1S methods as well. In order to do so we randomly down-sampled the SRSLY prep data to 5-fold coverage to adhere to the sequencing depth gathered from both the TaKaRa and Swift preps. The results are detailed in Additional file 4.

### Assessing the features of SRSLY

#### 5-prime and 3-prime overhangs

**Fig. 3:**
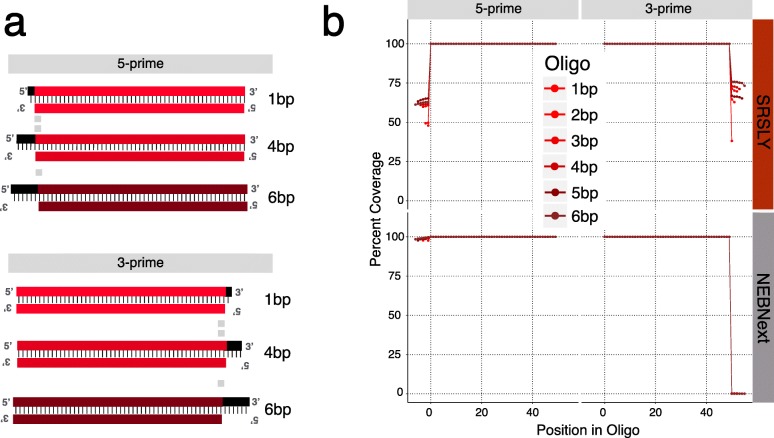
Coverage of duplexed oligos containing single-stranded overhangs for SRSLY and NEBNext. (**a**) Cartoon schematic of duplexed synthetic oligos – one blunt end, an identifiable 50 nt complementary region, and an overhang of specific length and type. (**b**) Average coverage per base across the length of all duplexed oligos for three technical replicates in 0 base coordinates for both SRSLY and NEBNext methods. Technical replicates were not statistically different from each other (Students t-test: SRSLY *p* = 0.714, NEBNext *p* = 0.985), error bars not shown for aesthetics. Each oligo sequenced > 5000 reads

Given the base composition differences in cfDNA at the 5-prime and 3-prime ends, we designed an experiment to test whether SRSLY and dsDNA library preparation methods, like NEBNext Ultra II, are altering (or not altering) input DNA fragments as we expect. We constructed pools of 12 synthetic duplexed oligos, at equimolar concentrations, each having a specific length and type (5-prime or 3-prime) overhang. Each duplex contains a 50 nucleotide (nt) core sequence, unique to each overhang type and has a common structure: blunt terminus on one side, and a 5-prime or 3-prime overhang of a specific length of random sequence (one to six nt) on the other side (Fig. 3a; Additional file 5).

We generated both SRSLY and NEBNext Ultra II libraries by spiking this pool of oligos into cfDNA extracts. From the sequencing data, we identified reads that originate from the oligo pool by mapping the libraries to a reference file containing the known unique 50 nt core sequences of each oligo. We then calculate the depth of coverage at every position for each oligo in the pool, including the overhangs. Since the duplexed oligos are comprised of two single-stranded molecules with one strand that is one to six nt longer than its complement, we expected the SRSLY method to yield sequence data with 100% coverage across the complementary region and 50% coverage across the overhangs. The results (Fig. 3b) confirm that SRSLY produces reduced coverage across the overhanging regions compared to the double-stranded regions of the synthetic oligos illustrating the method’s ability to yield stranded data that accurately characterizes the input DNA. By contrast, the libraries produced by NEB Ultra II demonstrate the expected result of end-polishing. Five-prime overhang are filled-in, resulting in almost full coverage on the *complementary strand* of molecules with known 5-prime overhangs. Three-prime exonuclease activity, on the other hand, causes nearly complete loss of the 3-prime overhang sequence when it is present.

#### Single-stranded oligo libraries

**Fig. 4:**
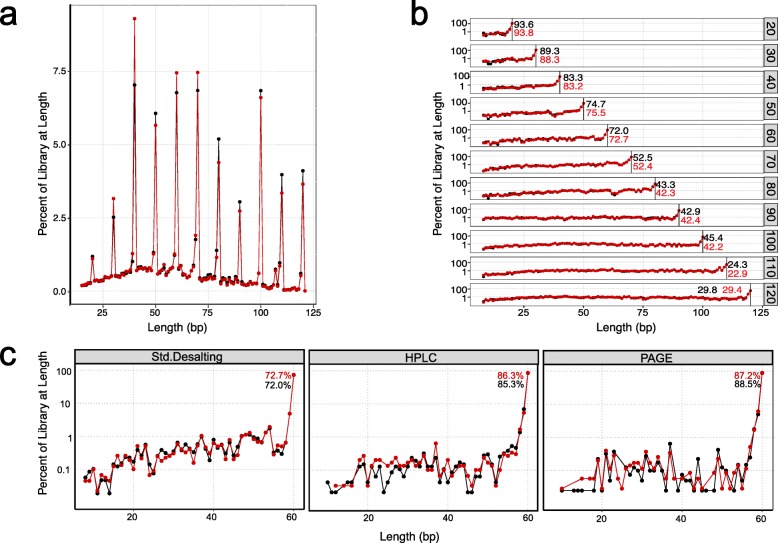
Single-stranded oligo analyses by the SRSLY method. Red and black lines and dots represent technical replicates (**a**) Insert distribution of equimolar pooled single-stranded oligo libraries. Oligos from 20 to 120 nt synthesized at 10 nt intervals were purified by standard desalting. Raw unfiltered sequencing data. (**b**) Mapped sequencing data for technical replicates separated by oligo. Represented as a function of oligo length. Black vertical bar and associated black and red numbers indicate percent of full-length product per oligo length present in the library pool. Each library was sequenced to a depth of ~ 100,000 read pairs (10,000 read pairs per oligo, excluding 20 and 30 nt lengths) (**c**) Effects for various purification methods on oligo purity as a function of oligo length for a 60 nt synthesized oligo. Associated black and red numbers indicate percent of full-length product per oligo. Data for the standard desalted 60 nt synthetic oligo pulled from (b)

To test the efficiency of SRSLY on a defined range of input DNA template lengths, we designed and ordered a set of 11 single-stranded oligos (standard desalt purification) of lengths ranging from 20 to 120 nucleotides at 10 nt length intervals (Additional file 6). We made a pool using equimolar concentrations of each and generated SRSLY libraries from this pool. Analysis of the proportion of template lengths from sequencing these libraries shows that the SRSLY protocol generates ssDNA libraries across this length range (Fig. 4a). As a control, we attempted to generate a NEBNext Ultra II libraries from this pool of single-stranded oligos. As expected, this protocol fails to generate any library at all using a template of exclusively single-stranded input DNA (libraries contained adapter dimers but no detectable yield at expected size distributions).

There were several noteworthy observations from the SRSLY data analysis. First, the shortest test oligos (20 and 30 nt length) were under-presented in the libraries. This is likely due to the bead clean-up step after the ligation, which has a known length bias against DNA oligos in this size range. We note that DNA fragments less than 30 nt are often difficult to map uniquely within genomes and are thus of less value, even when present in actual cfDNA samples. Second, there is some variation in library conversion efficiency amongst the longer (> = 40 nt) test oligos. We suspect that this variation is likely due to subtle biases in our test oligos, which are a single, fixed sequence for each length. Finally, we observe a continuous background fraction of oligo lengths that do not correspond to the input oligo lengths. In fact, we observe at least some reads of *every* length between 20 and 120.

To test whether these reads of unexpected length are due to truncated and incomplete oligo synthesis or due to labile breakage of our longer single-stranded oligos we mapped all the reads in the SRSLY libraries to their respective oligo reference (Fig. 4b, Additional file 7). Truncation products were present for each oligo. These truncated DNA fragments have lengths that are nearly uniformly distributed across the length of the oligo. The fraction of correct, full-length read mapping to each oligo decreases as a function of oligo length. We hypothesize that these two observations demonstrate the limits of the phosphoroamadite method of oligo synthesis. These observations are consistent with a model wherein nucleotide incorporation is less than 100% efficient in each chemical cycle of base addition.

To test whether SRSLY can assess the purity of oligos subjected to various purification methods, we ordered a 60 nt oligo purified using three common schemes: standard desalt, HPLC, and PAGE purification. We constructed SRSLY libraries, in duplicate, using the 60 nt oligo from all three purification methods. Mapping the sequence data to the 60 nt reference sequence (Fig. 4c) showed that the proportion of reads attributed to the expected full length sequence increases in both the HPLC and PAGE purified oligo libraries while truncation products, defined as reads at lengths shorter than 60 nt, decrease compared to the libraries generated from standard desalt oligos. These results are consistent with the expected quality of each purification method based on phosphoramidite synthesis (Integrated DNA Technologies Product Literature) and indicate that SRSLY can be used as a simple and sensitive assay to determine the purity of chemically synthesized DNA oligos.

### Analysis of SRSLY cfDNA libraries

The majority of cfDNA fragments derive from DNA wrapped around a nucleosome, a configuration that protects the DNA from nuclease degradation during cell death. Thus, the genomic map positions of cfDNA fragments can be used to infer the positions of histones and other DNA binding proteins in the tissues that have given rise to a population of cfDNA molecules [16]. Single-stranded DNA library methods, like SRSLY, retain the native ends of cfDNA fragments and are thus maximally useful for inferring the positions of histones and other DNA-binding proteins insofar as these proteins protect the DNA from endonuclease activity. We combined SRSLY data from our two healthy individuals (H-69 and H-81), to obtain 30-fold average genome coverage. From these data, we explored the ability of SRSLY libraries to reveal aspects of the positioning of nucleosomes and other DNA-binding proteins.

**Fig. 5:**
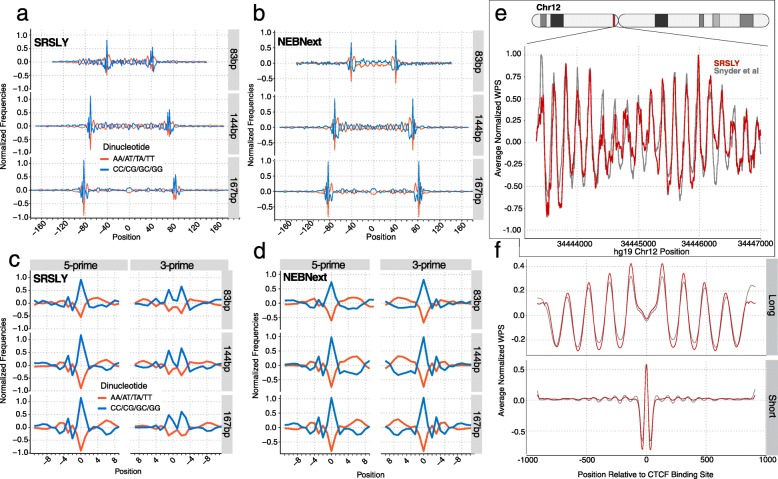
cfDNA analysis. (**a**) Normalized genomic dinucleotide frequencies as a function of read length for SRSLY data for three discrete fragment lengths including 100 bp ± the read mapped coordinates. Read midpoint is centered at 0. Negative numbers denote genomic regions upstream (5-prime) of the midpoint and positive numbers denote genomic regions downstream (3-prime) of the midpoint. Input data is from the combined H-69 and H-81 SRSLY datasets. (**b**) Same as (a) except for NEBNext data. (**c**) Normalized genomic dinucleotide frequency as a function of read length for SRSLY data for the termini of three discrete fragment lengths including a 9 bp region into the read (positive numbers) and 10 bp outside the read (negative numbers). Read start and end coordinates are centered on 0. Input data is from the combined H-69 and H-81 SRSLY datasets. (**d**) Same as (c) except for NEBNext data. (**e**) Normalized WPS values (120 bp window; 120–180 bp fragments) for SRSLY data compared to sample CH01 [16] at the same pericentromeric locus on chromosome 12 used to initially showcase WPS. (**f**) Average normalized WPS score within ±1 kb of annotated CTCF binding sites for long fragment length binned data (120 bp window, 120–180 bp fragments) and short fragment length binned data (16 bp window, 35–80 bp fragments) for SRSLY data compared to sample CH01 [16]

It is well established that nucleosome positioning is at least partially encoded by the genome [36, 38]. For DNA bound to histones, A/T dinucleotides are favored when the minor grove faces towards the histone and G/C dinucleotides are favored when the minor grove faces outwards. Therefore, when analyzed in aggregate, DNA fragments originating from nucleosome protected DNA should contain an oscillating pattern of an A/T rich and G/C depleted region directly followed by a G/C rich and A/T depleted region within captured fragments, compared to the surrounding genomic regions. To test whether we observed this oscillation pattern in our SRSLY data we examined the A/T and G/C genomic dinucleotide in molecules of three fragment lengths, 167, 144, and 83 bp, including bases 100 nts upstream and downstream of each of the three read lengths (Fig. 5a). We centered each on the midpoint of the sequence. As noted, 167 bp corresponds to the length of DNA wrapped around the nucleosome core particle plus the associated linker region, 144 bp represents the length of DNA wrapped around the nucleosome core particle only, and 83 bp may represents a degradation product originating from nucleosome-associated DNA.

Consistent with previous results from other ssDNA methods, we observe an oscillation enrichment for A/T and G/C dinucleotides within the sequenced molecule length compared to the surrounding genomic regions [16, 22]. We also observe a strong oscillation signal for ~ 55 bp upstream of the 83 bp fragment length indicating that these molecules are likely derived from degraded nucleosomal associated DNA. We also observe this dinucleotide oscillation within the defined fragment lengths for the NEBNext dsDNA method as well (Fig. 5b). However, we do not observe the upstream oscillation signal in the 83 bp fragment length for the NEBNext data. This may be due to low recovery of short fragments in the dsDNA preparation methods or other differences in the ability of dsDNA preps to convert fragmented or nicked DNA into sequencing libraries.

An additional feature of dinucleotide-mediated histone wrapping is that DNase I mediated nicking occurs when the minor grove is accessible [36, 39–41]. This phenomenon leads to a specific enrichment for G/C dinculeotides at the terminal ends of nucleosome-associated fragments (Figs. 5a-d). Due to the dsDNA end-polishing step, the terminal profile of the 5-prime and 3-prime ends in NEBNext data are mirror images of each other (Fig. 5d). The fact that the dinucleotide frequency at 3-prime termini differs considerably between SRSLY and NEBNext suggests that a substantial population of diverse overhangs occurs in a population of nucleosome-associated cfDNA fragments (Fig. 5c,d).

Next, we looked at nucleosome positioning using the window protection score (WPS) [16]. The WPS is a measure of whether a position in the genome tends to be protected from endonuclease activity or enriched for endonuclease activity. It is a function of how many reads span the given position (and thus were not cut) versus how many reads begin or end at that position (and thus were cut). We calculated the normalized WPS using SRLSY data at a region comprised of well-positioned nucleosomes on chromosome 12. Comparing our WPS results with previously published results using an alternative ssDNA library protocol, we observe good concordance with respect to the location of the peaks and troughs (Fig. 5c; Overall Pearons Correlation: r = 0.80, *p* < 0.0001) [16, 36, 42].

We performed a second WPS validation of our SRSLY data by calculating normalized WPSs for fragments whose lengths fall into a long-sized bin (120–180 bp, the range of fragments lengths presumed to derived from histone protection) and a short-sized bin (35–80 bp, presumed to be enriched for fragments protected by other DNA-binding proteins) within 1 kb upstream or 1 kb downstream of experimentally determined binding sites for the transcription factor CTCF (Fig. 5d). CTCF is a DNA-binding protein the occludes histones where it is bound and organizes histone positioning upstream and downstream [16, 43, 44]. Consistent with the previously described pattern, we find that the long fragment WPS shows a depression centered at the putative CTCF binding site (position 0) and oscillation patterns extending outward in both directions at a periodicity of ~ 180 bp indicating well-positioned nucleosomes. The short fragment results show a strong peak centered at the putative CTCF binding site, presumably due to CTCF-protection from endonuclease activity. Upstream and downstream, the smaller amplitude oscillations are consistent with the absence of DNA-binding proteins other than nucleosomes.

## Discussion

Although the merits of single-stranded NGS library approaches have been well described elsewhere [16, 25], there are no simple, efficient, and widely accessible protocols for making ssDNA libraries. While SRSLY generates sequencing library molecules from single-stranded DNA fragments, it can be used for DNA that is either single-stranded, double-stranded or a combination of the two. Thus, it is a drop-in replacement for a wide variety of NGS applications. It offers a fast, simple, ligation-based DNA library preparation that relies only on ubiquitously available reagents and an improved splint adapter design to create complex sequencing ready libraries in less than 3 h. The enhanced dual splint adapters allow SRSLY to benefit from the ligation efficiency of T4 DNA ligase. Because the adapter and splint oligos contain ligation-blocking modifications on every end except the ones where ligation should occur, the ligation reaction has been optimized for complete ligation. Unlike previous methods that use T4 DNA ligase to bind splint adapters to single-stranded template, our improved design eliminates the creation of a second strand via extension required for the final ligation, further reducing the possibility of introducing sequencing artifacts or errors into the preparation method.

We present validation of the SRSLY method via comparison to traditional dsDNA library preparation methods and a commercially available ssDNA preparation method showing that SRSLY produces sequencing libraries with uniform coverage, higher complexity, and base composition similar to those of the widely used NEBNext Ultra II kit. In contrast to dsDNA library methods, SRSLY converts a larger proportion of short DNA fragments into sequencing library molecules and retains the native termini of all input DNA fragments. On average, SRSLY cfDNA libraries are comprised of ~ 8% of DNA fragments in the 30–100 bp size bin compared to < 1% for the NEBNext kit. Like others, we have also observed increases in subnucleosomal DNA content in plasma from cancer patients (data not shown) [45–47]. Notably, the proportion of short fragments recovered by SRSLY can be modulated by altering the clean-up step following index PCR (Additional file 8).

We also demonstrated the utility of SRSLY’s native termini retention using two groups of synthetic control oligos. By calculating the depth of coverage at each position for synthetic duplex oligos containing single-stranded DNA overhangs we showed that SRSLY is able to retain strand information from dsDNA and a more accurate characterization of the template molecules. By generating SRSLY libraries from synthetic single-stranded oligos we showed that SRLSY can assay synthetic oligos for artifacts of incomplete synthesis. While this approach is straight-forward and powerful, we note that our assay can only report on DNA fragments with 5-prime and 3-prime ends with the capacity to be ligated. Further exploration may be warranted for more complete analysis of synthetic or biologically-derived DNA fragments that lack ligatable ends.

cfDNA fragments are in many ways an ideal substrate for demonstrating the benefits of ssDNA library preps [16]. cfDNA is often present in low quantities and is comprised of short and often-nicked DNA fragments. Further, the precise mapping positions of cfDNA reads, powered by ssDNA library prep, can reveal an added dimension to sequence-based DNA analysis like the positions of nucleosomes or DNA-binding proteins [48]. We find that the base composition surrounding fragmentation points in cfDNA differs between the 5-prime and 3-prime ends. This observation is consistent with the hypothesis that many or most cfDNA fragments are not blunt-ended since in that case every 5-prime end would have a corresponding 3-prime end. Further analysis of data generated from SRSLY may reveal further details of the nature of the overhangs present in cfDNA molecules and perhaps the identities of the active nucleases that generate them.

SRSLY is a simple and versatile tool for the preparation of sequencing libraries from fragmented single-stranded DNA. With only slight modifications, SRSLY could be adapted for use for other DNA sources besides cfDNA. The DNA present in FFPE samples is notoriously difficult for high-quality sequencing library preparation because it is fragmented and nicked. In preliminary tests, we have generated high-quality libraries from DNA recovered from FFPE samples and plan to adapt the protocol to the special challenges of this important input source. Another example is using SRSLY in a modified protocol for strand-accurate RNAseq libraries. Most methods for converting RNA into sequencing libraries either do not retain information about which DNA strand was transcribed or required additional steps to mark and destroy or mark and recover one strand of a double-stranded cDNA product. We have performed proof-of-concept experiments wherein first-strand reverse transcriptase products are used directly as input for SRSLY. These preliminary experiments, using a protocol much simpler than those currently available, generate RNAseq libraries retaining strand information as expected and are high complexity.

## Conclusions

We have developed a fast, simple, and efficient ligation-based single-stranded DNA library preparation method engineered to produce complex NGS libraries from one nanogram of DNA without altering the native ends of template molecules. Our method, called SRSLY (Single-Reaction Single-stranded LibrarY), requires no exotic reagents, can be completed in 2.5 h, and works in a one-step combined phosphorylation/ligation reaction that simultaneously prepares template DNA molecules for ligation without end-polishing while ligating Illumina adapters. SRLSY produces libraries with uniform coverage, higher complexity, and base composition similar to libraries generated by the widely used NEBNext Ultra II kit, all while retaining an increased proportion of short fragment length DNA. While we focus on cfDNA as input for SRSLY, we believe the benefits of SRSLY are easily expandable to other applications.

## Methods

### Molecular methods

#### Human cell-free DNA preparation

Whole blood from healthy donors was commercially purchased from Stanford Blood Center, Palo Alto, CA. Donors were deidentified, no biographic or clinical information was provided to Claret Biosciences LLC. Blood plasma was extracted from whole blood by spinning the blood collection tubes at 1800 g for 10 min at 4 °C. Without disturbing the cell layer, the supernatant was transferred to microfuge tubes under sterile conditions in 2 ml aliquots and spun again at 16000 g for 10 min at 4 °C to remove cell debris. cfDNA was prepared from 4 ml plasma using the Circulating Cell-free DNA kit (Qiagen Technologies) following manufacturer’s protocol. Concentration of the purified cell-free DNA (cfDNA) was measured using the Quant-iT high sensitivity dsDNA Assay Kit and a Qubit Fluorometer (ThermoFisher Scientific). cfDNA size distribution was analyzed using TapeStation and associated D5000 or D1000 high sensitivity products (Agilent).

#### Synthetic oligo preparation

Double-stranded synthetic oligos (Additional file 5) were designed using a random sequence generator at 50% GC content; sequences matching any known organism in public databases were removed. Each dsDNA oligo (*n* = 12) is a unique 50 nt sequence of double-stranded DNA with one blunt-end, and one 3-prime or 5-prime single-stranded overhang of random sequence, 1 to 6 nucleotides in length. Oligos were synthesized using standard desalting purification and duplexed by Integrated DNA Technologies (IDT); all random nucleotides were ‘hand-mixed’ to reduce synthesis bias. Control oligos were pooled together in an equimolar ratio for SRSLY library preparation.

Single-stranded synthetic oligos (Additional file 6) were generated in the same way as the double-stranded control oligos. Unless otherwise noted, oligos were synthesized using standard desalting purification for ssDNA oligos 20–80 nt in length and Ultramer purification for ssDNA oligos 90–120 nt in length.

#### SRSLY adapter preparation

The forward (P5) SRSLY adapter as well as the reverse (P7) SRSLY adapter are both double-stranded splint adapters. The forward SRSLY adapter contains a 5-prime overhang in the splint portion of the adapter and a free 3-prime OH end on the ligating end; all other ends contain ligation and/or extension blocking modifications. The reverse SRSLY adapter contains a 3-prime overhang in the splint portion of the adapter and is 5-prime phosphorylated for ligation; all other ends contain ligation and/or extension blocking modifications (Additional file 9)**.** The SRSLY adapters are synthesized using standard desalting purification and duplexed by Integrated DNA Technologies (IDT). Working stocks of the adapters are made by diluting the adapters in TE + 50 mM NaCl.

#### SRSLY library preparation

1 ng of purified cfDNA or 5 ng of synthesized oligos, as measured by the Quant-iT, was combined with 10 mM Tris pH 8.0 and 8 ng of ET SSB (New England Biolabs) in a 22 μl denaturation reaction, on ice. The reaction was placed in a thermocycler preheated to 95 °C, incubated for 3 min, and then cold shocked on ice for at least 2 min. On ice, 1 pmol of the forward and 1 pmol of the reverse SRSLY adapters were added to the denaturation reaction, as well as PEG-8000, T4 DNA ligase Buffer, T4 PNK, and T4 DNA ligase (all New England Biolabs) to a final volume of 50 μl. PEG-8000 was added to a final concentration of 18.5% v/v. T4 DNA ligase buffer was added to a final concentration of 1X. T4 PNK and T4 DNA ligase were added to a final concentration of 10 units and 800 units, respectively. This ligation reaction was incubated at 37 °C for one hour and purified using the MinElute PCR Purification Kit (Qiagen) and manufacturer’s instructions with the following changes: The initial binding spin was performed at 6000 rpm on a desktop centrifuge. The wash spin was repeated for a total of two wash spins and both washes were performed at 6000 rpm. The DNA was eluted in 15 μl 10 mM Tris pH 8.0.

SRSLY libraries were indexed for sequencing by combining the purified ligated DNA with 1x Kapa HiFi HotStart ReadyMix (Roche) and 2 mM final concentration of universal primer and 2 mM final concentration of an index primer in a 50 μl reaction and amplified using the following thermal cycling conditions: 3 min at 98 °C for initial denaturation followed by 10 cycles at 98 °C for 20 s, 68 °C for 30 s, 72 °C for 30 s, and finally an elongation step of 1 min at 72 °C. After index PCR, SRSLY libraries were purified with a 1.2x AMPure clean (Beckman Coulter) and eluted in 20 μl og 10 mM Tris pH 8.0. Final molarity estimates were calculated using fragment length distribution and dsDNA concentration (Agilent Tapestation 4200 and Qubit Fluorometric Quantitation unit).

#### NEBNext ultra II library preparation

1 ng of purified cfDNA or 5 ng of synthesized oligos, as measured by the Quant-iT, was taken through library preparation (end-polishing, adapter ligation, index PCR) as outlined in the NEBNext Ultra II manual using the supplied reagents, recommended AMPure cleanup ratios, and recommended index PCR cycles.

#### Sequencing

All cfDNA libraries were sequenced on an Illumina® HiSeqX at a 2 × 151 read length by Fulgent Genetics. All synthetic oligo libraries were sequenced on an in-house Illumina® MiSeq benchtop sequencer at a read length of 2 × 151 bp following manufacturer’s instructions.

### Informatic methods

#### Read processing

Sequencing data was first aligned to the PhiX genome using bwa mem [49] with default parameters. Reads that mapped to PhiX were discarded. Next we simultaneously removed adapter sequences and merged the reads as is standard practice in studies where short template molecules are expected [50]. This process consisted of collapsing forward and reverse reads into single sequences, based on sequence similarity, while trimming ends of reads that match known Illumina adapter sequences using SeqPrep (https://github.com/jstjohn/SeqPrep). Merged reads that remained after filtering were aligned to either the hg19 human reference genome (Additional files 1,2) downloaded from the UCSC genome browser [51], or to a custom fasta file corresponding to the synthesized oligo sequence (Additional file 7). We used bwa aln and bwa sampe [52]{Li, 2009 #24} with default parameters for alignment and mapping. Mapping rates, for human libraries, were determined from samtools flagstat. Duplicate reads were then removed using samtools rmdup.

#### QC metrics

For most analyses bam files from individual libraries of same preparation method and same cfDNA extract were merged into sample- and library-specific bam files using samtools merge prior to analysis. For insert length distribution of merged reads, for the same preparation method and cfDNA extract insert length information was parsed from the bam files of individual libraries that were generated using samtools view -q20 -f66 and combined using a concatenate command. Frequency of reads per length was calculated and plotted as the percent reads of total library. Normalized genome coverage was extracted from down-sampled merged duplicate removed bam files using samtools view -s such that all libraries had the same number of mapped reads. Data was obtained by pipping downsampled bam files from samtools view -q20 -b into bedtools genomecov. Preseq complexity estimates were obtained by combining only 3 libraries for each cfDNA input sample per library preparation method prior to downsampling in order to not artificially inflate the complexity of SRSLY, which had more libraries per cfDNA extract than NEBNext Ultra II. Libraries combined for SRSLY H-69 were: SR-01, SR-02, SR-03. Libraries combined for SRSLY H-81 were: SR-06, SR-07, SR-08. Libraries combined for NEBNext Ultra II for H-69 and H-81 were NEB-01-03A and NEB-04-06A, respectively. After combining and downsampling the combined bam files to 100 M merged read-pairs, complexity estimates and extrapolation were performed using preseq lcextract [53]. GC coverage was obtained from down-sampled merged duplicate removed bam files utilizing Picard Tools (Broad Institute) CollectGCBiasMetrics. For each library type, fragment terminal nucleotide analysis was done by calculating the proportion of each base i.e. the base composition, at every position for a region spanning from − 2 to + 34 bases on both reads of a fragment. The base composition per position was normalized with the mode for that base along the length of the region and log-2 transformed. The normalized, log-transformed proportions were calculated for both library types, for both reads and plotted. All plots were generated in R utilizing ggplot2.

#### Synthetic oligo analysis

Double-stranded synthetic oligo sequencing coverage at each position in the oligo was determined utilizing a custom script akin to samtools depth and plotted in R utilizing ggplot2 as a function of percent across the length of the oligos in 0 base coordinates.

Fragment length analysis of single-stranded synthetic oligos was conducted analogous to that for cfDNA.

#### Biological analysis of cfDNA

For dinucleotide frequency calculations merged bam files from combined H-69 and H-81 libraries for each library preparation method were parsed using samtools view -bh -F 0X10 -m -M -q 20 to extract forward reads of specific insert lengths: 167 bp (chromatosome-wrapped DNA length), 144 bp (core particle-wrapped DNA length, and 83 bp (a shorter DNA length that occurs as a peak in Fig. 2a). For each insert length, the dinucleotide counts around both fragmentation points were estimated using a custom python script for all 16 2-mer combination for either a 100 bp or 11 bp window, where 100 bp or 11 bp of genomic context at both 5-prime and 3-prime fragmentation points were added respectively. For the data generated with a 100 bp flanking window on both ends, the overlapping regions (which justifiably had the same counts) were removed. The data was normalized using a median filter and dinucleotide frequency was plotted for weak (AA/AT/TA/TT) vs strong (CC/CG/GC/GG) dinucleotide interaction such that the center of the insert was at 0 and the regions upstream of the fragmentation point had negative values and downstream had positive values. For the data generated with a 11 bp flanking window, the data was normalized with a median filter and dinucleotide frequencies of weak vs strong dinucleotide were plotted for 5-prime and 3-prime ends using R.

WPS scores were calculated in the manner previously described [16]: The WPS score for each position in the genome was determined by collecting the reads which align in a window around that position, 120 bp in the case of large fragment analysis and 35 bp in the case of short fragment analysis. The score was calculated as follows: Every time an insert starts or end in that window, one is subtracted from the score. If an insert does not start or end in that window, but aligns to it nevertheless, one is added to the score. The normalized WPS score was calculated by taking the WPS scores over non-overlapping 1000 bp segments and adjusting to a median score of zero by subtracting the median WPS score. The scores were then smoothed by the Savitzky–Golay filter: second-order polynomials were fitted to median-adjusted scores over a 21 bp window at each position. The smoothed score is the value of that polynomial at that position. The Average WPS score is calculated over a set of regions of equal length by calculating the mean of the WPS scores over each position in each of the regions in our set, where position 1 is the first nucleotide of each region in our set, position 2 is the second nucleotide in each region, etc. CTCF sites were chosen in a method similar to what was described previously [16]. A bed file containing a list of putative TF binding sites was downloaded from the JASPAR2018 table(hub_186875_JasparTFBS) from the UCSC Genome Browser Table Browser into a bed file and filtered to include only CTCF sites. These sites were compared with CTCF ChIP-Seq data from 19 cell lines [54]. Putative binding sites with overlapping ChIP-Seq peaks in all 19 cell lines were used for further analysis.

## Additional Files

**Additional file 1: Table S1.** SRSLY human cfDNA libraries NGS statistics. (docx 15 kb)

**Additional file 2: Table S2**. All ofther human cfDNA libraries NGS statistics. (docx 16 kb)

**Additional file 3: Figure S1.** Insert distribution for replicate libraries for H-69 and H-81. (docx 303 kb)

**Additional file 4: Figure S2.** Standard NGS metrics for merged reads from SRSLY, TaKara SMARTer, and Swift 1S libraries from healthy human cfDNA extracts H-69 and H-81

**Additional file 5: Table S3.** Synthetic duplexed oligos sequences. (docx 14 kb)

**Additional file 6: Table S4.** Synthetic single-stranded oligos sequences. (docx 14 kb)

**Additional file 7: Table S5.** Synthetic single-stranded oligo raw read counts. (docx 15 kb)

**Additional file 8: Figure S3.** Effect of post index PCR DNA purification on SRSLY fragment length retention. (docx 156 kb)

**Additional file 9: Table S6.** SRSLY adapter design. (docx 22 kb)

## Data Availability

The sequencing data sets in this manuscript were deposited in the National Center for Biotechnology Information Sequence Read Archive (http://ncbi.nlm.nih.gov/sra) under BioProject ID: PRJNA545528.

## Abbreviation

bp: Base-pair
cfDNA: Cell-free DNA
ctDNA: Circulating-tumor DNA
dsDNA: Double-stranded DNA
FFPE: Formalin-fixed paraffin-embedded
NGS: Next-Generation Sequencing
nt: Nucleotide
SRSLY: Single Reaction Single-stranded Library
SSB: Single-stranded binding protein
ssDNA: Single-stranded DNA
WPS: Window protection score

## References

[1] Goodwin S, McPherson JD, McCombie WR (2016). Coming of age: ten years of next-generation sequencing technologies. Nat Rev Genet.

[2] Meyer, M. & Kircher, M. Illumina sequencing library preparation for highly multiplexed target capture and sequencing. Cold Spring Harbor protocols 2010, pdb prot5448, doi:10.1101/pdb.prot5448 (2010). 10.1101/pdb.prot5448 20516186

[3] Bentley DR (2008). Accurate whole human genome sequencing using reversible terminator chemistry. Nature.

[4] Adey A (2010). Rapid, low-input, low-bias construction of shotgun fragment libraries by high-density in vitro transposition. Genome Biol.

[5] Bennett, E. A. et al*.* Library construction for ancient genomics: single strand or double strand? Biotechniques 56, 289–290, 292–286, 298, passim, doi:10.2144/000114176 (2014). 10.2144/000114176 24924389

[6] Dabney J (2013). Complete mitochondrial genome sequence of a middle Pleistocene cave bear reconstructed from ultrashort DNA fragments. Proc Natl Acad Sci U S A.

[7] Meyer M (2012). A high-coverage genome sequence from an archaic Denisovan individual. Science.

[8] Gansauge MT, Meyer M (2013). Single-stranded DNA library preparation for the sequencing of ancient or damaged DNA. Nat Protoc.

[9] Aravanis AM, Lee M, Klausner RD (2017). Next-generation sequencing of circulating tumor DNA for early Cancer detection. Cell.

[10] Agardh E (2015). Genome-wide analysis of DNA methylation in subjects with type 1 diabetes identifies epigenetic modifications associated with proliferative diabetic retinopathy. BMC Med.

[11] De Vlaminck I (2013). Temporal response of the human virome to immunosuppression and antiviral therapy. Cell.

[12] Fan HC, Blumenfeld YJ, Chitkara U, Hudgins L, Quake SR (2008). Noninvasive diagnosis of fetal aneuploidy by shotgun sequencing DNA from maternal blood. Proc Natl Acad Sci U S A.

[13] Jiang P (2015). Lengthening and shortening of plasma DNA in hepatocellular carcinoma patients. Proc Natl Acad Sci U S A.

[14] Sun K (2015). Plasma DNA tissue mapping by genome-wide methylation sequencing for noninvasive prenatal, cancer, and transplantation assessments. Proc Natl Acad Sci U S A.

[15] Jahr S (2001). DNA fragments in the blood plasma of cancer patients: quantitations and evidence for their origin from apoptotic and necrotic cells. Cancer Res.

[16] Snyder MW, Kircher M, Hill AJ, Daza RM, Shendure J (2016). Cell-free DNA comprises an in vivo nucleosome footprint that informs its tissues-of-origin. Cell.

[17] Lo YM (2010). Maternal plasma DNA sequencing reveals the genome-wide genetic and mutational profile of the fetus. Sci Transl Med.

[18] Murtaza M (2013). Non-invasive analysis of acquired resistance to cancer therapy by sequencing of plasma DNA. Nature.

[19] Newman AM (2014). An ultrasensitive method for quantitating circulating tumor DNA with broad patient coverage. Nat Med.

[20] Newman AM (2016). Integrated digital error suppression for improved detection of circulating tumor DNA. Nat Biotechnol.

[21] Tie J (2015). Circulating tumor DNA as an early marker of therapeutic response in patients with metastatic colorectal cancer. Ann Oncol.

[22] Wu DC, Lambowitz AM (2017). Facile single-stranded DNA sequencing of human plasma DNA via thermostable group II intron reverse transcriptase template switching. Sci Rep.

[23] Mouliere F (2011). High fragmentation characterizes tumour-derived circulating DNA. PLoS One.

[24] Quake S (2012). Sizing up cell-free DNA. Clin Chem.

[25] Burnham P (2016). Single-stranded DNA library preparation uncovers the origin and diversity of ultrashort cell-free DNA in plasma. Sci Rep.

[26] Gansauge MT (2017). Single-stranded DNA library preparation from highly degraded DNA using T4 DNA ligase. Nucleic Acids Res.

[27] Raine A, Manlig E, Wahlberg P, Syvanen AC, Nordlund J (2017). SPlinted ligation adapter tagging (SPLAT), a novel library preparation method for whole genome bisulphite sequencing. Nucleic Acids Res.

[28] Turchinovich A (2014). Capture and amplification by tailing and switching (CATS). An ultrasensitive ligation-independent method for generation of DNA libraries for deep sequencing from picogram amounts of DNA and RNA. RNA Biol.

[29] Wu J, Dai W, Wu L, Wang J (2018). SALP, a new single-stranded DNA library preparation method especially useful for the high-throughput characterization of chromatin openness states. BMC Genomics.

[30] Wu Jian, Dai Wei, Wu Lin, Li Weiwei, Xia Xinyi, Wang Jinke (2019). Decoding genetic and epigenetic information embedded in cell free DNA with adapted SALP‐seq. International Journal of Cancer.

[31] Soltis DA, Uhlenbeck OC (1982). Isolation and characterization of two mutant forms of T4 polynucleotide kinase. J Biol Chem.

[32] Wang LK, Lima CD, Shuman S (2002). Structure and mechanism of T4 polynucleotide kinase: an RNA repair enzyme. EMBO J.

[33] Wang LK, Shuman S (2001). Domain structure and mutational analysis of T4 polynucleotide kinase. J Biol Chem.

[34] Kuhn H, Frank-Kamenetskii MD (2005). Template-independent ligation of single-stranded DNA by T4 DNA ligase. FEBS J.

[35] Kostyuk S (2015). GC-rich extracellular DNA induces oxidative stress, double-Strand DNA breaks, and DNA damage response in human adipose-derived Mesenchymal stem cells. Oxidative Med Cell Longev.

[36] Gaffney DJ (2012). Controls of nucleosome positioning in the human genome. PLoS Genet.

[37] Harshman SW, Young NL, Parthun MR, Freitas MA (2013). H1 histones: current perspectives and challenges. Nucleic Acids Res.

[38] Satchwell SC, Drew HR, Travers AA (1986). Sequence periodicities in chicken nucleosome core DNA. J Mol Biol.

[39] Boyle AP (2008). High-resolution mapping and characterization of open chromatin across the genome. Cell.

[40] Cousins DJ (2004). Redefinition of the cleavage sites of DNase I on the nucleosome core particle. J Mol Biol.

[41] Segal E (2006). A genomic code for nucleosome positioning. Nature.

[42] Valouev A (2011). Determinants of nucleosome organization in primary human cells. Nature.

[43] Fu Y, Sinha M, Peterson CL, Weng Z (2008). The insulator binding protein CTCF positions 20 nucleosomes around its binding sites across the human genome. PLoS Genet.

[44] Ong CT, Corces VG (2014). CTCF: an architectural protein bridging genome topology and function. Nat Rev Genet.

[45] Jiang P, Lo YMD (2016). The long and short of circulating cell-free DNA and the ins and outs of molecular diagnostics. Trends Genet.

[46] Lapin M (2018). Fragment size and level of cell-free DNA provide prognostic information in patients with advanced pancreatic cancer. J Transl Med.

[47] Schwarzenbach H, Hoon DS, Pantel K (2011). Cell-free nucleic acids as biomarkers in cancer patients. Nat Rev Cancer.

[48] Sanchez C, Snyder MW, Tanos R, Shendure J, Thierry AR (2018). New insights into structural features and optimal detection of circulating tumor DNA determined by single-strand DNA analysis. NPJ Genom Med.

[49] Li, H. Aligning sequence reads, clone sequences and assembly contigs with BWA-MEM. arXiv preprint arXiv*:1303.3997* (2013).

[50] Kircher M (2012). Analysis of high-throughput ancient DNA sequencing data. Methods Mol Biol.

[51] Kent WJ (2002). The human genome browser at UCSC. Genome Res.

[52] Li H, Durbin R (2009). Fast and accurate short read alignment with burrows-wheeler transform. Bioinformatics.

[53] Daley T, Smith AD (2013). Predicting the molecular complexity of sequencing libraries. Nat Methods.

[54] Wang H (2012). Widespread plasticity in CTCF occupancy linked to DNA methylation. Genome Res.

