# Supplementary material for: A ligation-based single-stranded library preparation method to analyze cell-free DNA and synthetic oligos
Source: BMC Genomics. 2019 Dec 27;20:1023. doi: 10.1186/s12864-019-6355-0 (PMC6935139; doi:10.1186/s12864-019-6355-0)
Supplement: Supplementary file 1 — Additional file 1: Table S1. SRSLY human cfDNA libraries NGS statistics. (docx 15 kb) [file 12864_2019_6355_MOESM1_ESM.docx]

**Additional file 4: Table S1.** SRSLY human cfDNA extract NGS statistics.

| **Library ID** | **cfDNA extract** | **Raw read pairs** | **Pass filter read pairs** | **Merged read pairs** | **Mapped read pairs** | **Duplicate read pairs** |
| --- | --- | --- | --- | --- | --- | --- |
| SR-01 | H-69 | 94,786,943 | 86,884,321 (91.7%) | 74,059,050 (85.2%) | 69,735,053 (77.7%) | 6,646,784 (9.5%) |
| SR-02 | H-69 | 94,297,123 | 89,775,122 (95.2%) | 75,364,887 (83.9%) | 74,408,496 (85.6%) | 7,342,661 (9.9%) |
| SR-03 | H-69 | 81,474,103 | 77,874,288 (95.6%) | 65,201,784 (83.7%) | 64,727,039 (83.1%) | 5,958,851 (9.2%) |
| SR-04 | H-69 | 98,450,841 | 90,642,659 (92.1%) | 76,197,502 (84.1%) | 74,101,090 (81.8%) | 6,981,901 (9.4%) |
| SR-05 | H-69 | 115,200,247 | 105,758,818 (91.8%) | 86,567,929 (81.9%) | 85,410,946 (80.8%) | 9,571,162 (11.2%) |
| All | H-69 | 484,209,257 | 450,935,208  (93.1%) | 377,391,152  (83.7%) | 368,382,624  (81.7%) | 36,501,359  (9.9%) |
| SR-06 | H-81 | 84,140,424 | 80,948,813 (96.2%) | 71,429,415 (88.2%) | 68,958,103 (85.2%) | 9,122,834 (13.2%) |
| SR-07 | H-81 | 74,670,157 | 71,559,425 (95.8%) | 63,111,643 (88.2%) | 61,087,692 (85.4%) | 7,380,490 (12.1%) |
| SR-08 | H-81 | 77,438,201 | 74,583,049 (96.3%) | 65,654,147 (88.0) | 63,686,313 (85.4%) | 8,372,356 (13.1%) |
| SR-09 | H-81 | 84,600,059 | 81,361,847 (96.2%) | 72,265,939 (88.8%) | 70,187,322 (86.3%) | 8,495,259 (12.1%) |
| SR-10 | H-81 | 77,177,608 | 74,493,904 (96.5%) | 66,365,109 (89.1%) | 64,450,156 (86.5%) | 8,256,944 (12.8%) |
| All | H-81 | 398,026,449 | 382,947,038  (96.2%) | 338,826,253  (88.5%) | 328,369,586  (85.7%) | 41,627,883  (12.7%) |
