# Supplementary material for: A ligation-based single-stranded library preparation method to analyze cell-free DNA and synthetic oligos
Source: BMC Genomics. 2019 Dec 27;20:1023. doi: 10.1186/s12864-019-6355-0 (PMC6935139; doi:10.1186/s12864-019-6355-0)
Supplement: Supplementary file 2 — Additional file 2: Table S2. All ofther human cfDNA libraries NGS statistics. (docx 16 kb) [file 12864_2019_6355_MOESM2_ESM.docx]

**Additional file 5: Table S2.** Comparison library preps human cfDNA extract NGS statistics.

| **Library ID** | **Preparation kit** | **cfDNA extract** | **Raw read pairs** | **Pass filter read pairs** | **Merged read pairs** | **Mapped read pairs** | **Duplicate read pairs** |
| --- | --- | --- | --- | --- | --- | --- | --- |
| NEB-01A | NEB Ultra II | S0069 | 56,804,742 | 56,581,216 (99.6%) | 47,014,204 (83.1%) | 49,075,274 (86.7%) | 4,286,898 (8.7%) |
| NEB-02A | NEB Ultra II | S0069 | 62,159,092 | 61,939,780 (99.6%) | 51,079,841 (82.5%) | 53,969,240 (87.1%) | 5,281,009 (9.8%) |
| NEB-03A | NEB Ultra II | S0069 | 54,665,148 | 54,415,552 (99.5%) | 44,943,923 (82.6%) | 47,100,881 (86.6%) | 3,917,226 (8.3%) |
| NEB-04A | NEB Ultra II | S0081 | 45,040,369 | 44,945,092 (99.8%) | 39,385,036 (87.6%) | 40,229,499 (89.5%) | 4,519,419 (11.2%) |
| NEB-05A | NEB Ultra II | S0081 | 40,276,528 | 40,208,800 (99.8%) | 34,083,417 (84.8%) | 35,894,023 (89.3%) | 3,951,972 (11.0%) |
| NEB-06A | NEB Ultra II | S0081 | 38,184,951 | 38,107,032 (99.8%) | 33,641,066 (88.3%) | 34,066,689 (89.4%) | 3,653,615 (10.7%) |
| NEB-01B | NEB Ultra II | S0069 | 145,586,438 | 145,391,68(99.9%) | 124,430,880  (85.6%) | 130,678,620 (89.9%) | 21,702,629 (16.6%) |
| NEB-02B | NEB Ultra II | S0069 | 167,080,644 | 166,862,822 (99.9%) | 141,337,108  (84.7%) | 150,007,151  (89.9%) | 27,522,314 (18.3%) |
| NEB-03B | NEB Ultra II | S0069 | 149,861,198 | 149,652,929 (99.9%) | 128,254,214 (85.7%) | 134,494,174 (89.9%) | 21,894,091 (16.3%) |
| NEB-04B | NEB Ultra II | S0081 | 135,165,144 | 134,915,960  (99.8%) | 117,427,639  (87.0%) | 121,157,380  (89.8%) | 20,317,676 (16.8%) |
| NEB-05B | NEB Ultra II | S0081 | 134,972,563 | 134,765,678  (99.8%) | 112,962,099  (83.8%) | 120,756,517  (89.6%) | 20,380,321 (16.9%) |
| NEB-06B | NEB Ultra II | S0081 | 118,888,514 | 118686258  (99.8%) | 104,471,069  (88.0%) | 106,579,341  (89.8%) | 17,229,569 (16.2%) |
| SWT-01A | Swift NGS 1S Accel | S0069 | 57,267,715 | 50,061,414  (87.4%) | 42,649,149  (85.2%) | 41,942,763  (83.8%) | 4,410,212  (8.8%) |
| SWT-02A | Swift NGS 1S Accel | S0069 | 56,758,713 | 53,102,126  (93.6%) | 45,451,975  (85.6%) | 44,812,574  (84.4%) | 4,538,421  (8.6%) |
| SWT-03A | Swift NGS 1S Accel | S0069 | 65,931,917 | 61,112,695  (92.7%) | 51,810,429  (84.8%) | 51,802,844  (84.8%) | 5,815,032  (9.5%) |
| SWT-01B | Swift NGS 1S Accel | S0081 | 46,707,495 | 45.667,114  (97.8%) | 41,471,348  (90.8%) | 39,862,279  (87.3%) | 5,161,043  (11.3%) |
| SWT-02B | Swift NGS 1S Accel | S0081 | 46,016,658 | 44,426,052  (96.5%) | 40,374,114  (90.9%) | 38,466,813  (86.6%) | 4,755,327  (10.7%) |
| SWT-03B | Swift NGS 1S Accel | S0081 | 40,339,362 | 38,931,088  (96.5%) | 35,423,108  (91.0%) | 33,807,212  (86.8%) | 3,917,002  (10.1%) |
| TKA-01A | TaKaRa ThruPLEX Plasma-Seq | S0069 | 70,159,339 | 69,775,464  (99.5%) | 59,077,765  (84.7%) | 59,758,638  (85.6%) | 6,339,914  (9.1%) |
| TKA-02A | TaKaRa ThruPLEX Plasma-Seq | S0069 | 72,536,063 | 72,129,821  (99.4%) | 60,625,330  (84.1%) | 61,509,949  (85.3%) | 6,299,368  (8.7%) |
| TKA-03A | TaKaRa ThruPLEX Plasma-Seq | S0069 | 63,650,274 | 63,315,928  (99.5%) | 53,369,955  (84.3%) | 54,307,565  (85.8%) | 5,227,134  (9.5%) |
| TKA-01B | TaKaRa ThruPLEX Plasma-Seq | S0081 | 46,630,408 | 46,455,843  (99.6%) | 41,764,957  (89.9%) | 41,107,671  (88.5%) | 5,220,268  (11.2%) |
| TKA-02B | TaKaRa ThruPLEX Plasma-Seq | S0081 | 46,385,711 | 46,171,497  (99.5%) | 41,478,478  (89.8%) | 40,770,738  (88.3%) | 4,723,961  (10.2%) |
| TKA-03B | TaKaRa ThruPLEX Plasma-Seq | S0081 | 44,421,901 | 44,254,484  (99.6%) | 40,012,865  (90.4%) | 39,169,322  (88.5%) | 4,961,394  (11.2%) |
