# Supplementary material for: A ligation-based single-stranded library preparation method to analyze cell-free DNA and synthetic oligos
Source: BMC Genomics. 2019 Dec 27;20:1023. doi: 10.1186/s12864-019-6355-0 (PMC6935139; doi:10.1186/s12864-019-6355-0)
Supplement: Supplementary file 3 — Additional file 3: Figure S1. Insert distribution for replicate libraries for H-69 and H-81. (docx 303 kb) [file 12864_2019_6355_MOESM3_ESM.docx]

**Additional file 2: Figure S2 – Insert distributions for replicate libraries for H-69 and H-81.** (**A**) Insert distribution for all libraries made for cfDNA extract H-69. (**B**) Insert distribution for all libraries made for cfDNA extract H-81.
