# Supplementary material for: A ligation-based single-stranded library preparation method to analyze cell-free DNA and synthetic oligos
Source: BMC Genomics. 2019 Dec 27;20:1023. doi: 10.1186/s12864-019-6355-0 (PMC6935139; doi:10.1186/s12864-019-6355-0)
Supplement: Supplementary file 4 — Additional file 4: Figure S2. Standard NGS metrics for merged reads from SRSLY, TaKara SMARTer, and Swift 1S libraries from healthy human cfDNA extracts H-69 and H-81 [file 12864_2019_6355_MOESM4_ESM.pdf]

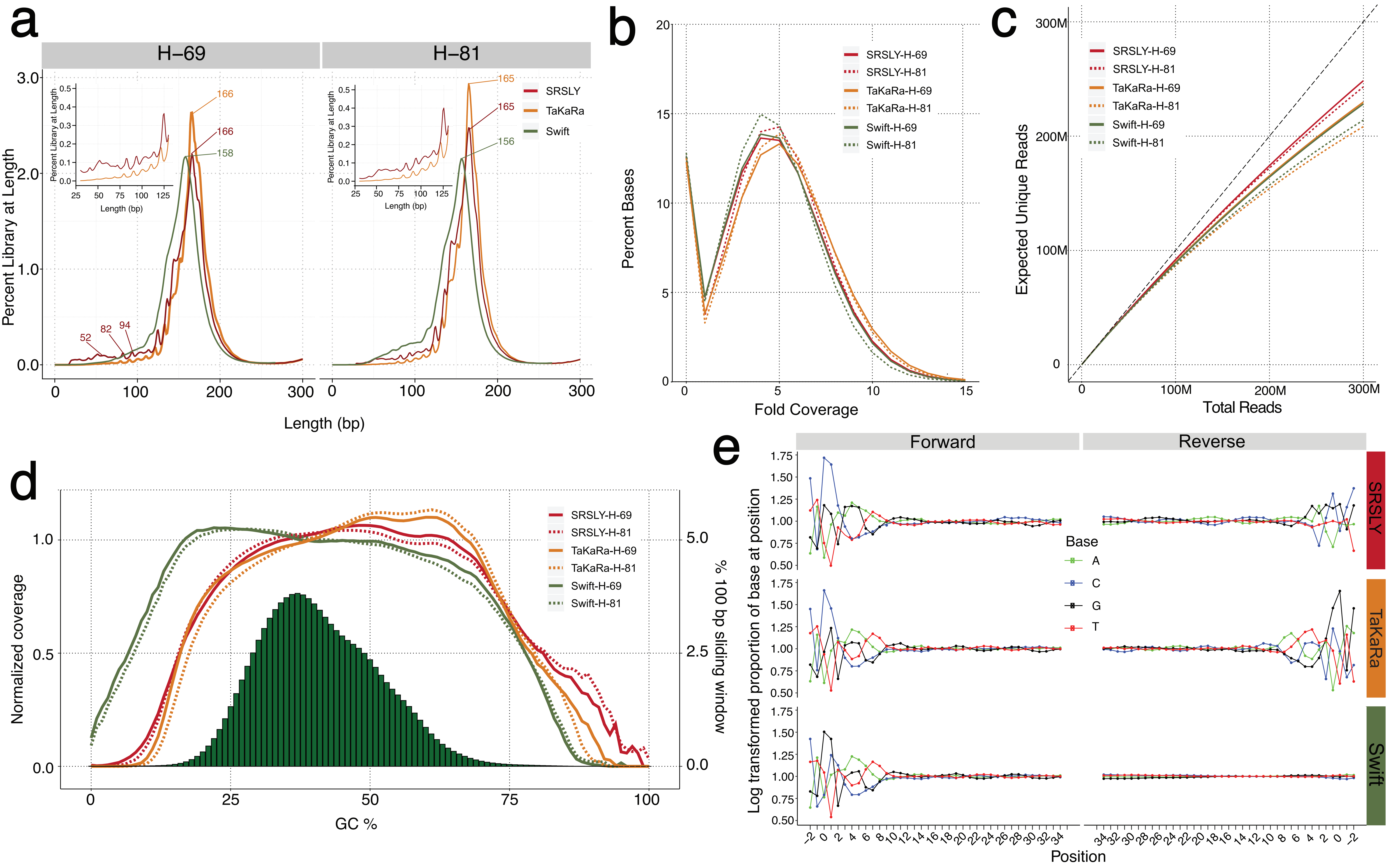

Additional file 4: Figure S2 - Standard NGS metrics for merged reads from SRSLY, TaKara SMARTer, and Swift 1S libraries from healthy human cfDNA extracts H-69 and H-81. Three, 1 nanogram libraries per cfDNA extract per library preparation method for both TaKaRa SMARTerÒ ThruPLEXÒ Plasma-Seq and Swift Bioscience Accel-NGSÒ 1S were combined and downsampled to 100 million merged read-pairs to create around 5-fold coverage (Additional file 2). Combined bam files for SRSLY libraries from both cfDNA extracts were downsampled to 100 million merged read-pairs to create a comparable dataset. Data from the Swift Bioscience Accel-NGSÒ 1S kit was preprocessed. The last 15 bases of the merged read-pairs (i.e. the first 15 bases from the start of read 2) were trimmed prior to mapping to remove the synthetic Adaptase tail, as per recommendations in Swift product literature (https://swiftbiosci.com/wp-content/uploads/2019/02/16-0853-Tail-Trim-Final-442019.pdf). Figures were created as described in the methods.

(a) Insert distribution plots for cfDNA extracts H-69 and H-81, respectively. TaKaRa data produces a similar distribution as the NEBNext Ultra II data (Figure 2). Due to data preprocessing, the main peak of the Swift data is artificially at a shorter length and the biologically relevant sawtooth pattern in fragments shorter than 130 bp is mostly lost. (b) Fold coverage by base percent across the human genome (hg19) for SRSLY, TaKaRa, and Swift based on 100 million merged read-pairs per cfDNA extract. All libraries produce similar fold-coverage and relatively uniform genomic coverage. (c) Preseq complexity estimate for SRSLY, TaKaRa, and Swift by cfDNA extract. All three methods produce high complexity libraries with SRSLY estimated complexity higher than TaKaRa or Swift for both cfDNA extracts. (d) Normalized coverage as a function of GC content over 100 bp sliding scale across the human genome for SRSLY, TaKaRa, and Swift by cfDNA extract. GC coverage for SRSLY and TaKaRa follow similar trends with TaKaRa having slightly higher coverage in genomic regions with 50% – 75% GC percent. Swift distribution is biased towards AT rich regions when compared to both SRSLY and TaKaRa data. (e) Normalized, log-transformed base composition at each position of read termini starting 2 bp upstream and extending to 34 bp downstream of read start site for combined cfDNA extracts for SRSLY, TaKaRa, and Swift. TaKaRa data reproduces the results seen by the NEBNext Ultra II data (Figure 2). Due to data preprocessing, the 3-prime signal (start of the reverse read) of the Swift data is lost. Also, the fragmentation biases around the forward read position 0 for the Swift data deviates for G and C bases from those observed in the SRSLY, TaKaRa, and NEBNext data.
