# Supplementary material for: A ligation-based single-stranded library preparation method to analyze cell-free DNA and synthetic oligos
Source: BMC Genomics. 2019 Dec 27;20:1023. doi: 10.1186/s12864-019-6355-0 (PMC6935139; doi:10.1186/s12864-019-6355-0)
Supplement: Supplementary file 5 — Additional file 5: Table S3. Synthetic duplexed oligos sequences. (docx 14 kb) [file 12864_2019_6355_MOESM5_ESM.docx]

| Overhang Type | Sequence 1 | Sequence 2 |  |
| --- | --- | --- | --- |
| 3’ 1bp | CCATACTGTGGTCGTCACCTATTACCCCGCGTAAAGGTAGGCTATGTCATN_1_ | ATGACATAGCCTACCTTTACGCGGGGTAATAGGTGACGACCACAGTATGG | |
| 3’ 2bp | GTGAATTGTTGATGTCCTGGGTGCCTCGTCCCAAAAGCTGTCCTCACGACN_2_ | GTCGTGAGGACAGCTTTTGGGACGAGGCACCCAGGACATCAACAATTCAC | |
| 3’ 3bp | GCTTCTCGAACCCGCGATCCGGCCGATCCGGCATAATGGGTTGATTTAGAN_3_ | TCTAAATCAACCCATTATGCCGGATCGGCCGGATCGCGGGTTCGAGAAGC | |
| 3’ 4bp | CGACACGGATATTCCATCAAGAGACGGGCCTATGGTCCCTGTGATGATGTN_4_ | ACATCATCACAGGGACCATAGGCCCGTCTCTTGATGGAATATCCGTGTCG | |
| 3’ 5bp | ACCTTGTGTGTTGCTGAAGCAAAGCCGCGTGACCGTTTTAACCAGCGAACN_5_ | GTTCGCTGGTTAAAACGGTCACGCGGCTTTGCTTCAGCAACACACAAGGT | |
| 3’ 6bp | ATTTTACCACGAGTTCCTTACGACGGCTGTGATGCCACGGTAGGCAGGTAN_6_ | TACCTGCCTACCGTGGCATCACAGCCGTCGTAAGGAACTCGTGGTAAAAT | |
| 5’ 1bp | N_1_CGCTTTACGGGTCCTGGGCCGGGGTGCGATACCTTGCAGAAATCGAGGCC | GGCCTCGATTTCTGCAAGGTATCGCACCCCGGCCCAGGACCCGTAAAGCG | |
| 5’ 2bp | N_2_AGGACTCTGCCGTCGACGAGTTCGTTAATTCACGGCATCACGTGCGTAGT | ACTACGCACGTGATGCCGTGAATTAACGAACTCGTCGACGGCAGAGTCCT | |
| 5’ 3bp | N_3_ACCTCCGTCGCGCTATGTTCTGTTGCATTCGACCTTCTCCGTTCTGTGGG | CCCACAGAACGGAGAAGGTCGAATGCAACAGAACATAGCGCGACGGAGGT | |
| 5’ 4bp | N_4_ACAAGAGGAGCATCCGTATTACCGCCTATATCGCCTACGTTTAGAGCATT | AATGCTCTAAACGTAGGCGATATAGGCGGTAATACGGATGCTCCTCTTGT | |
| 5’ 5bp | N_5_GTAAATCCCACACAGCTGTCGGCTTATATGGTCATTGGACGGCGTAATAG | CTATTACGCCGTCCAATGACCATATAAGCCGACAGCTGTGTGGGATTTAC | |
| 5’ 6bp | N_6_CCAGACAGCCATAGAGGTTACAAGCATAGCAATTTGCATCAGTTCGCAGA | TCTGCGAACTGATGCAAATTGCTATGCTTGTAACCTCTATGGCTGTCTGG | |

**Additional file 5: Table S3.** Synthetic duplexed oligos sequences
