# Supplementary material for: A ligation-based single-stranded library preparation method to analyze cell-free DNA and synthetic oligos
Source: BMC Genomics. 2019 Dec 27;20:1023. doi: 10.1186/s12864-019-6355-0 (PMC6935139; doi:10.1186/s12864-019-6355-0)
Supplement: Supplementary file 6 — Additional file 6: Table S4. Synthetic single-stranded oligos sequences. (docx 14 kb) [file 12864_2019_6355_MOESM6_ESM.docx]

| **Oligo** | **Sequence (5’ -> 3’)** |
| --- | --- |
| **20mer** | GTA AAG GTA GGC TAT GTC AT |
| **30mer** | GTG CCT CGT CCC AAA AGC TGT CCT CAC GAC |
| **40mer** | GCT TCT CGA ACC CGC GAT CCG GCC GAT CCG GCA TAA TGG G |
| **50mer** | CGA CAC GGA TAT TCC ATC AAG AGA CGG GCC TAT GGT CCC TGT GAT GAT GT |
| **60mer** | ATT TTA CCA CAC CTT GTG TGT TGC TGA AGC AAA GCC GCG TGA CCG TTT TAA CCA GCG AAC |
| **70mer** | CCA TTC GGG CAT AAT ATG AAC TAT ACG CAG CTT ATC CCG GGC CCG TAA CAA ACA ATT TGC GTG AGG TAT G |
| **80mer** | GTC CCA CTC AGA GAA TTA GCA GCC CTG GTC TAG CGA GGG ATG CCG CTT AGC GTC GGT TGA ATT TCG CTG CAC TAC AGA CG |
| **90mer** | CGC TTT ACG GGT CCT GGG CCG GGG TGC GAT ACC TTG CAG AAT CTG CGC CTC TTG GTG GCG CCC CAT CAG TAG TGT CTA CAC GGG CGC TGT |
| **100mer** | GTA AAT CCC ACA CAG CTG TCG GCT TAT ATG GTC ATT GGA CGG CGT AAT AGA CAA GAG GAG CAT CCG TAT TAC CGC CTA TAT CGC CTA CGT TTA GAG CAT T |
| **110mer** | GGT TCC TAA CAG GTG ATT ACC AGT GCA GTT AGC CAT TTA TCC TCG TCA AAA AGC CAC GTT CCA GAC AGC CAT AGA GGT TAC AAG CAT AGC AAT TTG CAT CAG TTC GCA GA |
| **120mer** | GAC GGC CCT AGT CTG CTT CTC GAG ACA ATC TGC TAG AAC TCG GAC GCC TCG CAC TGT ACT GAT GCA TGG TCC GTA ATC GAG GTG AAA ACT ACA CGG TAT GAC ATC AGC GAT AAC TGG TTT |

**Additional File 6: Table S4.** Synthetic single-stranded oligo sequences
