# Supplementary material for: A ligation-based single-stranded library preparation method to analyze cell-free DNA and synthetic oligos
Source: BMC Genomics. 2019 Dec 27;20:1023. doi: 10.1186/s12864-019-6355-0 (PMC6935139; doi:10.1186/s12864-019-6355-0)
Supplement: Supplementary file 7 — Additional file 7: Table S5. Synthetic single-stranded oligo raw read counts. (docx 15 kb) [file 12864_2019_6355_MOESM7_ESM.docx]

**Additional file 7: Table S5.** Synthetic single-stranded oligo raw read counts

| **Library ID** | **Oligo length** | **Raw mapped reads** | **Library ID** | **Oligo Length** | **Raw mapped reads** |
| --- | --- | --- | --- | --- | --- |
| Replicate 1 | 20 bp | 1241 | Replicate 2 | 20 bp | 876 |
| Replicate 1 | 30 bp | 2864 | Replicate 2 | 30 bp | 2918 |
| Replicate 1 | 40 bp | 9802 | Replicate 2 | 40 bp | 10144 |
| Replicate 1 | 50 bp | 9340 | Replicate 2 | 50 bp | 6481 |
| Replicate 1 | 60 bp | 10437 | Replicate 2 | 60 bp | 8761 |
| Replicate 1 | 70 bp | 15275 | Replicate 2 | 70 bp | 13055 |
| Replicate 1 | 80 bp | 14465 | Replicate 2 | 80 bp | 9531 |
| Replicate 1 | 90 bp | 8229 | Replicate 2 | 90 bp | 5678 |
| Replicate 1 | 100 bp | 18801 | Replicate 2 | 100 bp | 14240 |
| Replicate 1 | 110 bp | 20577 | Replicate 2 | 110 bp | 13938 |
| Replicate 1 | 120 bp | 17567 | Replicate 2 | 120 bp | 12004 |
| Replicate 1 | All | 128598  (96.47%) | Replicate 2 | All | 97626  (96.24%) |
| Replicate 1 | 60 bp HPLC | 4686 | Replicate 2 | 60 bp HPLC | 2966 |
| Replicate 1 | 60 bp PAGE | 4038 | Replicate 2 | 60 bp PAGE | 3520 |
