# Supplementary material for: A ligation-based single-stranded library preparation method to analyze cell-free DNA and synthetic oligos
Source: BMC Genomics. 2019 Dec 27;20:1023. doi: 10.1186/s12864-019-6355-0 (PMC6935139; doi:10.1186/s12864-019-6355-0)
Supplement: Supplementary file 8 — Additional file 8: Figure S3. Effect of post index PCR DNA purification on SRSLY fragment length retention. (docx 156 kb) [file 12864_2019_6355_MOESM8_ESM.docx]

**Additional file 8: Figure S3 – Effect of post index PCR DNA purification on SRSLY fragment length retention.** SRSLY libraries for cfDNA H-69 were purified using either a 1.2x or 1.5x DNA purification bead volume:Index PCR reaction volume ratio. Recovery of <100 bp fragments changed from 9.3% to 14.7% for the higher ratio from the lower ratio
