# Supplementary material for: A ligation-based single-stranded library preparation method to analyze cell-free DNA and synthetic oligos
Source: BMC Genomics. 2019 Dec 27;20:1023. doi: 10.1186/s12864-019-6355-0 (PMC6935139; doi:10.1186/s12864-019-6355-0)
Supplement: Supplementary file 9 — Additional file 9: Table S6. SRSLY adapter design. (docx 22 kb) [file 12864_2019_6355_MOESM9_ESM.docx]

| **Adapter** | **Sequence 1 (Adapter)** | **Sequence 2 (Splint)** |
| --- | --- | --- |
| **Forward (P5)** | /5AmMC12/ACACTCTTTCCCTACACGACGCTCTTCCGATCT | /5AmMC6/NNNNNNNAGATCGGAAGAGCGTCGTGTAGGGAAAGAGTGT/3AmMO/ |
| **Reverse (P7)** | /5Phos/AGATCGGAAGAGCACACGTCTGAACTCCAGTCA/3ddC/ | /5AmMC12/GTGACTGGAGTTCAGACGTGTGCTCTTCCGATCTNNNNNNN/3AmMO/ |

**Additional file 9: Table S6.** SRSLY adapter design
